# Natural convection of Nanoliquid from a Cylinder in Square Porous Enclosure using Buongiorno’s Two-phase Model

**DOI:** 10.1038/s41598-019-57062-x

**Published:** 2020-01-10

**Authors:** Abeer Alhashash

**Affiliations:** Department of Mathematics, College of Science, Jouf University, P.O. Box 2014 Sakaka, Saudi Arabia

**Keywords:** Applied mathematics, Fluid dynamics

## Abstract

Natural convection of nanoliquid in a square porous enclosure has been studied using non homogeneous two-phase Buongiorno’s model. The outer of enclosure has cold temperature and a circular cylinder is put at the center. A finite heated segment is located on the top cylinder surface which is otherwise insulated. The momentum in the porous layer is modeled applying the Brinkman-Forchheimer equations. The analysis are conducted in the following interval of the associated groups: the portion of heated surface (5% ≤ *H* ≤ 100%), the concentration (0.0 ≤ *ϕ* ≤ 0.04), the Darcy number, 10^−5^ ≤ *D*_*a*_ ≤ 10^−2^ and the cylinder size, (0.15 ≤ *R* ≤ 0.25). The minimum heat transfer rate of the active surface were obtained at location *ξ* = 90°. In general, the ratio of the heat transfer per unit area of the heat source decreases as the length of the heated surface increases. The heat transfer rate is intensified for the half thermally active surface and high value of Darcy number at higher nanoparticles concentration.

## Introduction

Management convection heat transfer becomes an important aspect in thermal device such as handled devices, electronic equipments and heat exchangers. With maximum loads often in the order of second and ever-small packages design of the handheld devices that restrict their surface temperatures. Heat exchangers may be grouped by configuration, transfer process, flow organization, etc. In industrial processes, another technique for enhancing the thermal performance is using porous medium.

One of the technique to improve heat transfer is to utilise porous medium with nanoliquid. A nanoliquid is called as a smart liquid with added metals of sizes less than 100 nm in classical heat transfer liquid such as water, glycol and oil^1^. studied a triangle porous enclosure filled with with nanoliquid where the heater attached to the wall. The conductive solid attached on the wall were considered by^2–6^. They investigated the effect of the wall conditions and nanoliquid properties on the overall heat transfer. Heated partially porous enclosure was studied by^7,8^ investigated the interaction between nanoparticles and magnetic field. Influence of internal chemical reaction in the porous layer was considered by^9^. The Tiwari and Das model with new formulation for the physical properties of the nanoliquid was applied by^10,11^. Systematic review of free convection in porous media with nanoliquid was given by^12,13^ studied transient convective flow in porous square enclosure having solid obstacle and nonuniform boundary condition^14^. Considered three heat equation model^15^. Investigated the influence of nonuniform side walls temperature on heat transfer characteristics of nanoliquid^16^. Considered hybrid nanoliquid or suspend more than one type of nanoparticles into the base liquid. They observed that decreasing of the Nusselt number is much higher for hybrid nanoliquid compared to the single nanoliquid. Recently^17,18^, considered the annulus porous enclosure with Darcy model and the influences of Lorentz forces are taken into account.

The above literatures review assume that liquid phase and the solid is in thermal equilibrium condition with the slip velocity between solid and the liquid is ignored. This approach is called as a single-phase model. The multi-phase model assumes the slip velocity between the liquid phase and the solid, so that the multi-phase nanoliquid model confirmed to be more realistic the Buongiorno’s multi-phase nanoliquid model together with the Brownian motion and thermophoresis effects were studied by^19^. They reported that the Nusselt number is increasing functions of the ration of heating intensity and thermophoresis parameters but decreasing functions of the the ratio of thermal diffusivity and Brownian motion parameters^20^. Included the dissipation and surface radiation parameters to the Darcy flow and found that the influence of viscous dissipation on the concentration patterns is fairly large^21^. Considered non-equilibrium heat equations and treat the porous media using the Brinkman model. They found that if the cylindrical heater is put at the below of the enclosure, the concentration distribution get homogeneous because of the agitation by powerful flow circulation^22^. Treated the porous media using the Brinkman–Forchheimer model and conclude that a proper selection of heater configuration and orientation angle combined with nanoliquid concentration and porosity parameters can extremely modify the heat transfer^23^. Found increasing the heat transfer parameter at the interface boost the heat transfer in the solid matrix but reduces the heat transfer in the nanoliquid^24^. Concluded that the hybrid nanofluids retard the flow circulation and the heat transfer performance later the increment of wall thickness, porosity, interface heat transfer coefficient can increase or decrease the flow circulation and the thermal performance^25^. Reported that the variability of porosity and the convection intensity indicated considerably effects on the Nusselt number and the concentration distribution^26^. Found that the presence of hybrid nanofluids increases the heat transfer performance over the plate. Recently^27^, observed that the fusion temperature is the important aspect on the thermal performance enhancement.

Free convection from a hot cylinder inserted in the porous enclosure were well studied in previous researches^28^ considered the bottom and vertical sides are insulated while the top wall is cold and found a small variation of the heat transfer by adjusting the cylinder size. The active cylinder embedded in the center of the deferentially heated enclosure studied by^29,30^ filled the porous enclosure with a water-based nanoliquid suspending Ag, Cu, Al_2_O_3_, or TiO_2_ solid nanoparticles. They searched the optimum cylinder radius and applied the Darcy flow with the Tiwari and Das nanoliquid models. The review of the reference indicates that nanoliquid considering the Buongiorno’s two-phase nanoliquid model incorporating the Brownian motion and thermophoresis forces has not received enough attention. The present work aims to investigated the free convection by heating cylinder surface having a constant heat flux inserted in the porous enclosure using Brinkman–Forchheimer model. The inner cylinder is heated partially with constant heat flux. Previously, the partial heating of the side or bottom walls were studied by^31–34^. The heat source size was found to modify the liquid flow and the thermal performance. The application of partial heating occurs in the cooling electronic device where the boards and component represent the finite heat source.

## Mathematical Formulation

**Figure 1:**
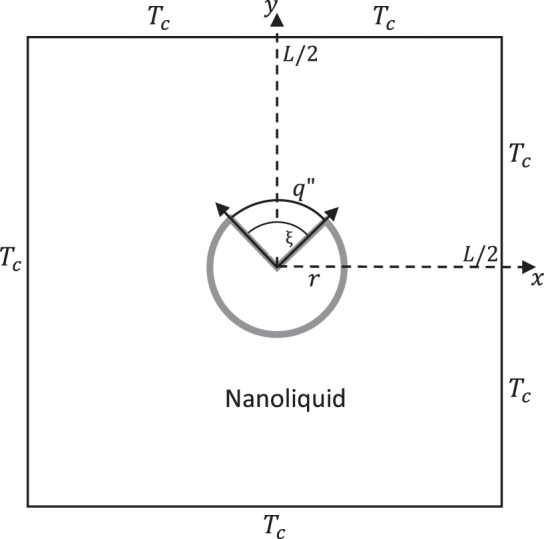
Schematic representation of the model.

Figure 1 sketch a system coordinate of a square porous enclosure with a cylinder. The enclosure were considered at a constant low temperature. The cylinder has radius *r* and put in the middle point of the square. A finite heated segment $\xi$ with constant heat flux is located on the top cylinder surface which is otherwise insulated. Under the effect of the gravitational acceleration, the cylinder and enclosure at different temperature gradient bring to a buoyancy problem. All of the surfaces are considered to be impermeable, the liquid in the enclosure is a water-based nanoliquid having Al_2_O_3_. The Boussinesq approximation is assumed valid for the density variation. Based on these considerations, the continuity, momentum, thermal energy, nanoparticles and nanoparticles mass flux equations can be stated as follows^35^:

1 $$\nabla \cdot {\bf{v}}=0,$$

2 $$\begin{array}{rcl}{\rho }_{nl}\frac{{\bf{v}}}{{\varepsilon }^{2}}\cdot \nabla {\bf{v}} & = & -\nabla p+\nabla \cdot \frac{({\mu }_{nl}\nabla {\bf{v}})}{\varepsilon }+{(\rho \beta )}_{nl}g\frac{\partial T}{\partial x}\\  &  & -\,\frac{150{\mu }_{nl}{(1-\varepsilon )}^{2}}{{d}^{2}{\varepsilon }^{3}}{\bf{v}}-\frac{262.5d{(1-\varepsilon )}^{2}}{{d}^{2}{\varepsilon }^{3}}|{\bf{v}}|{\bf{v}},\end{array}$$

3 $$\frac{{(\rho {C}_{p})}_{nl}}{\varepsilon }{\bf{v}}\cdot \nabla T=-\,\nabla \cdot ({k}_{nl}\nabla T)-{C}_{p,p}{J}_{p}\cdot \nabla T,$$

4 $${\bf{v}}\cdot \nabla \varphi =-\,\frac{\varepsilon }{{\rho }_{p}}\nabla \cdot {J}_{p},$$

5 $${J}_{p}=-\,{\rho }_{p}{D}_{B}\nabla \varphi -{\rho }_{p}{D}_{T}\nabla T$$

symbol *g* is the gravity acceleration, $\varphi$ is the concentration and ${J}_{p}$ is the nanoparticles mass flux. ${D}_{B}$ is the Brownian diffusion coefficient, ${D}_{B}=\frac{{k}_{b}T}{3\pi {\mu }_{l}{d}_{p}}$. ${D}_{T}$ is the thermophoretic diffusion coefficient, ${D}_{T}=0.26\frac{{k}_{l}}{2{k}_{l}+{k}_{p}}\frac{{\mu }_{l}}{{\rho }_{l}T}\varphi$. The specific heat capacitance of the nanoliquid ${(\rho {C}_{p})}_{nl}$ is stated as

6 $${(\rho {C}_{p})}_{nl}=(1-\varphi ){(\rho {C}_{p})}_{l}+\varphi {(\rho {C}_{p})}_{p}.$$

The diffusivity of the nanoliquid *α*_*nl*_ is defined as

7 $${\alpha }_{nl}=\frac{{k}_{nl}}{{(\rho {C}_{p})}_{nl}}$$

The density of the nanoliquid ${\rho }_{nl}$ can be determined

8 $${\rho }_{nl}=(1-\varphi ){\rho }_{l}+\varphi {\rho }_{p}$$

The thermal expansion coefficient of the nanoliquid *β*_*nl*_ is formulated as:

9 $${(\rho \beta )}_{nl}=(1-\varphi ){(\rho \beta )}_{l}+\varphi {(\rho \beta )}_{p}$$

The dynamic viscosity ratio of nanoliquid in the the room temperature was given by^36^ as follows.

10 $$\frac{{\mu }_{nl}}{{\mu }_{l}}=\frac{1}{[1-34.87{({d}_{p}/{d}_{l})}^{-0.3}{\varphi }^{1.03}]}$$

The conductivity ratio of nanoliquid is given by^36^ as follows:

11 $$\frac{{k}_{nl}}{{k}_{l}}=1+4.4{{\rm{Re}}}_{B}^{0.4}{{\rm{\Pr }}}^{0.66}{(\frac{T}{{T}_{fr}})}^{10}{(\frac{{k}_{p}}{{k}_{l}})}^{0.03}{\varphi }^{0.66}.$$

with

12 $${{\rm{Re}}}_{B}=\frac{{\rho }_{l}{u}_{B}{d}_{p}}{{\mu }_{l}}.$$

13 $${u}_{B}=\frac{2{k}_{b}T}{\pi {\mu }_{l}{d}_{p}^{2}}.$$

With ${k}_{b}$ is the average relative kinetic energy of nanoparticles in the liquid with the temperature of the liquid. It is called as The Boltzmann constant, defined to be exactly ${k}_{b}=1.380648\times {10}^{-23}\,(J/K)$. ${l}_{l}=0.17\,{\rm{nm}}$ is the average path of liquid. ${d}_{l}$ is the molecular diameter of base fluid defined as^36^

14 $${d}_{l}=\frac{6M}{N\pi {\rho }_{l}}.$$

With $M$ is the molecular mass of the water, $N$ is the number of constituent nanoparticles that are contained in one mole and ${\rho }_{l}$ is the density of the water. Consequently and basing on water as a host liquid, the value of ${d}_{l}$ is evaluated as:

15 $${d}_{l}={(\frac{6\times 0.01801528}{6.022\times {10}^{23}\times \pi \times 998.26})}^{1/3}=3.85\times {10}^{-10}\,{\rm{m}}.$$

Introducing the non-dimensional variables as follow:

16 $$\begin{array}{l}X=\frac{x}{L},Y=\frac{y}{L},V=\frac{vL}{{\nu }_{l}},P=\frac{p{L}^{2}}{{\rho }_{nl}{\nu }_{l}^{2}},{\varphi }^{\ast }=\frac{\varphi }{\phi },{D}_{B}^{\ast }=\frac{{D}_{B}}{{D}_{B0}},{D}_{T}^{\ast }=\frac{{D}_{T}}{{D}_{T0}},\\ \Theta =\frac{T-{T}_{c}}{\Delta T},R=\frac{r}{L},\Delta T=\frac{q\mbox{''}L}{{k}_{l}}\end{array}$$

This then produces the dimensionless continuity, momentum, heat transfer and nanoparticle equations are:

17 $$\frac{\partial U}{\partial X}+\frac{\partial V}{\partial Y}=0$$

18 $$\begin{array}{rcl}(\frac{{\rho }_{nl}}{{\rho }_{l}})\frac{U}{{\varepsilon }^{2}}\frac{\partial U}{\partial X}+(\frac{{\rho }_{nl}}{{\rho }_{l}})\frac{V}{{\varepsilon }^{2}}\frac{\partial U}{\partial Y} & = & -(\frac{{\rho }_{nl}}{{\rho }_{l}})\frac{\partial P}{\partial X}\\  &  & +\,(\frac{{\mu }_{nl}}{{\mu }_{l}\varepsilon })(\frac{{\partial }^{2}U}{\partial {X}^{2}}+\frac{{\partial }^{2}U}{\partial {Y}^{2}})\\  &  & +\,(\frac{{\rho }_{nl}}{{\rho }_{l}}\frac{{\mu }_{nl}}{{\mu }_{l}})\frac{U}{Da}\\  &  & -\,(\frac{{\rho }_{nl}}{{\rho }_{l}}\frac{{\mu }_{nl}}{{\mu }_{l}})\frac{{C}_{F}}{{\Pr }}\frac{\sqrt{{U}^{2}+{V}^{2}}}{\sqrt{Da}}U\end{array}$$

19 $$\begin{array}{rcl}(\frac{{\rho }_{nl}}{{\rho }_{l}})\frac{U}{{\varepsilon }^{2}}\frac{\partial V}{\partial X}+(\frac{{\rho }_{nl}}{{\rho }_{l}})\frac{V}{{\varepsilon }^{2}}\frac{\partial V}{\partial Y} & = & -(\frac{{\rho }_{nl}}{{\rho }_{l}})\frac{\partial P}{\partial Y}\\  &  & +\,(\frac{{\mu }_{nl}}{\varepsilon {\mu }_{l}})(\frac{{\partial }^{2}V}{\partial {X}^{2}}+\frac{{\partial }^{2}V}{\partial {Y}^{2}})\\  &  & +\,[(\frac{{(\rho \beta )}_{nl}}{{(\rho \beta )}_{l}})\frac{1}{{\Pr }}Ra\Theta ]\\  &  & +\,(\frac{{\rho }_{nl}}{{\rho }_{l}}\frac{{\mu }_{nl}}{{\mu }_{l}})\frac{V}{Da}\\  &  & -\,(\frac{{\rho }_{nl}}{{\rho }_{l}}\frac{{\mu }_{nl}}{{\mu }_{l}})\frac{{C}_{F}}{{\Pr }}\frac{\sqrt{{U}^{2}+{V}^{2}}}{\sqrt{Da}}V\end{array}$$

20 $$\begin{array}{rcl}\frac{U}{\varepsilon }\frac{\partial \Theta }{\partial X}+\frac{V}{\varepsilon }\frac{\partial \Theta }{\partial Y} & = & \frac{{(\rho {C}_{p})}_{l}}{{(\rho {C}_{p})}_{nl}}\frac{{k}_{nl}}{{k}_{l}}\frac{1}{{\rm{\Pr }}}(\frac{{\partial }^{2}\Theta }{\partial {X}^{2}}+\frac{{\partial }^{2}\Theta }{\partial {Y}^{2}})\\  &  & +\,\frac{{(\rho {C}_{p})}_{l}}{{(\rho {C}_{p})}_{nl}}\frac{{D}_{B}^{\ast }}{{\rm{\Pr }}\,Le}(\frac{\partial \Phi }{\partial X}\frac{\partial \Theta }{\partial X}+\frac{\partial \Phi }{\partial Y}\frac{\partial \Theta }{\partial Y})\\  &  & +\,\frac{{(\rho {C}_{p})}_{l}}{{(\rho {C}_{p})}_{nl}}\frac{{D}_{T}^{\ast }}{{\rm{\Pr }}\,Le{N}_{BT}}[{(\frac{\partial \Theta }{\partial X})}^{2}+{(\frac{\partial \Theta }{\partial Y})}^{2}],\end{array}$$

21 $$\frac{U}{\varepsilon }\frac{\partial \Phi }{\partial X}+\frac{V}{\varepsilon }\frac{\partial \Phi }{\partial Y}=\frac{{D}_{B}^{\ast }}{Sc}(\frac{{\partial }^{2}\Phi }{\partial {X}^{2}}+\frac{{\partial }^{2}\Phi }{\partial {Y}^{2}})+\frac{{D}_{T}^{\ast }}{Sc{N}_{BT}}(\frac{{\partial }^{2}\Theta }{\partial {X}^{2}}+\frac{{\partial }^{2}\Theta }{\partial {Y}^{2}})$$

Symbols ${D}_{B0}=\frac{{k}_{b}{T}_{c}}{3\pi {\mu }_{l}{d}_{p}}$ is the reference Brownian diffusion coefficient, ${D}_{T0}=0.26\frac{{k}_{l}}{2{k}_{l}+{k}_{p}}\frac{{\mu }_{l}}{{\rho }_{l}\theta }\phi$ is the reference thermophoretic diffusion factor, *Sc* is the ratio of kinematic viscosity and mass diffusivity (Schmidt number), ${N}_{BT}=\phi {D}_{B0}{T}_{c}/({D}_{T0}\Delta T)$ is the diffusivity ratio parameter, $Le={k}_{l}/{(\rho {C}_{p})}_{l}\phi {D}_{B0}$ is Lewis number, $Ra=g{\rho }_{l}{\beta }_{l}\Delta T{L}^{3}/({\mu }_{l}{\alpha }_{l})$ is the Rayleigh number, $Da=\frac{K}{{L}^{2}}$ is the Darcy number and ${\rm{\Pr }}={\nu }_{l}/{\alpha }_{l}$ is the Prandtl number, ${C}_{F}=1.75/\sqrt{150}$ is the Forchheimer constant, $K={\varepsilon }^{2}{D}_{p}^{2}/(150{[1-\varepsilon ]}^{2})$ is the permeability of the porous medium, *D*_*p*_ is spherical beads diameter^37^ and ${k}_{{\rm{eff}}}=\varepsilon +(1-\varepsilon ){k}_{s}/{k}_{l}$ is the effective porous medium conductivity^38^. The non-dimensional boundary conditions are:

22 $$U=V=0,\,\frac{\partial {\varphi }^{\ast }}{\partial n}=\frac{\partial \Theta }{\partial n}=0\,{\rm{on}}\,{\rm{the}}\,{\rm{insulated}}\,{\rm{cylinder}}\,{\rm{surface}},$$

23 $$U=V=0,\,\frac{\partial {\varphi }^{\ast }}{\partial n}=-\,\frac{{D}_{T}^{\ast }}{{D}_{B}^{\ast }}\cdot \frac{1}{{N}_{BT}}\cdot \frac{\partial \Theta }{\partial n},\frac{\partial \Theta }{\partial n}=-\,1\,{\rm{on}}\,{\rm{the}}\,{\rm{heated}}\,{\rm{cylinder}}\,{\rm{surface}},$$

24 $$U=V=0,\,\frac{\partial {\varphi }^{\ast }}{\partial n}=-\,\frac{{D}_{T}^{\ast }}{{D}_{B}^{\ast }}\cdot \frac{1}{{N}_{BT}}\cdot \frac{\partial \Theta }{\partial n},\,\Theta =0\,{\rm{on}}\,{\rm{the}}\,{\rm{bottom}},{\rm{right}},{\rm{top}}\,{\rm{and}}\,{\rm{left}}\,{\rm{walls}},$$

A reference pressure is required to determine a unique pressure field. Any other point in the domain with a preference value could be took as a reference pressure. Here, the bottom right point of the boundary is null and selected as a pressure reference. This adopted value does not influence the solution at all. The reference pressure level is equivalent to 1 ATM in absolute pressure for the default reference pressure.

The local heat transfer for the surface with constant heat flux is

25 $$N{u}_{nl}=\frac{q\mbox{''}L}{{k}_{l}(T-{T}_{c})}=\frac{{k}_{nl}}{{k}_{l}}\frac{1}{\Theta }$$

Finally, the averaged heat transfer at the heated surface is defined as

26 $${\overline{Nu}}_{nl}=\frac{1}{\xi 2\pi RH}\,{\int }_{-\xi /2}^{\xi /2}\,Nu(\xi ){\rm{d}}\xi $$

where $\xi$ is the angular position and *H* is the portion of the heated segment defined as:

27 $$H=\frac{\xi }{360}$$

## Solution Method

The governing equations and the boundary condition was solved numerically by the finite element method (FEM). Divisions of the calculation domain into finite elements where the close form of each of the temperature (isotherms), velocity (streamfunctions) and nanoparticle distribution (iso concentrations) variables were given by^39^.

Computational domain element is generated by triangular shapes. For each of the dependent variables inside the calculation domain, high-orders and low-orders of triangular Lagrange finite elements were utilized. For the continuity, momentum and energy equations, evaluating of residuals was conducted by replacing the close form into the governing equations. A Newton method is utilized to approximate the nonlinear part of the momentum equations.

**Figure 2:**
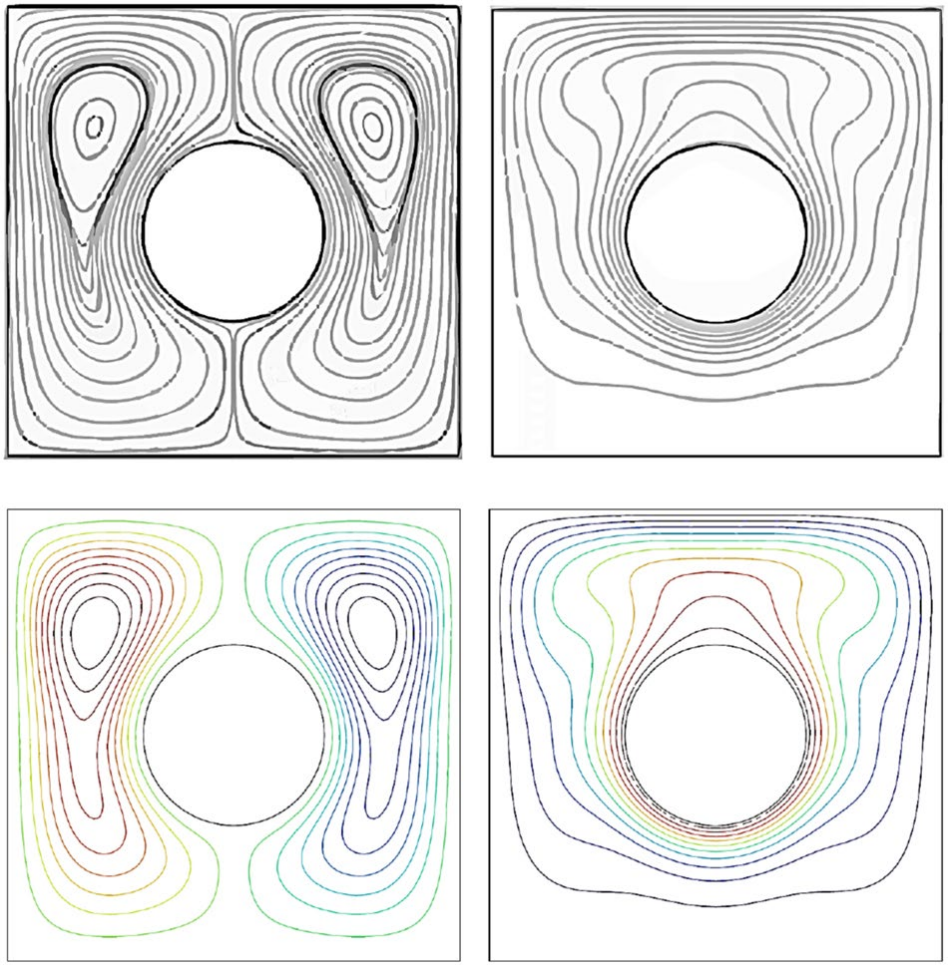
Validation of computed streamlines and isotherms of the present work (bottom) with that of^40^ (top) at $Ra={10}^{5}$, $R=0.2$, $H=100 \%$ and $\varphi =0.0$.

In the process of mathematical calculation, the mesh generation on the domain is made by using non-uniform triangles. Various grid sensitivity checks were conducted to evaluate the sufficiency of the grid scheme and to make certain of the results are accurate. In the tests, considering the case at point $(x=-\,0.25,y=0.35)$ for the $H=37.5 \%$, $R=0.2$, $\varphi =0.03$, $Da=0.01$ and $Ra={10}^{5}$ as tabulated in Table 1. Small quantity variation of the selected variable is observed from the extra fine grid size. Considering the time of computation, then the extra fine grid was chose for all the calculations conducted in this simulation. As a verification, the computations for the streamline and isotherms match well with those reported by^40^ at $Ra={10}^{5}$, $R=0.2$, $H=100 \%$ and $\varphi =0.0$ for nonporous case, $\varepsilon =0.99$, $Da={10}^{7}$ as displayed in Fig. 2.

**Table 1:** Grid sensitivity tests at coordinate $(x=-\,0.25,y=0.35)$ for the $H=37.5 \%$, $R=0.2$, $\varphi =0.03$, $Da=0.01$ and $Ra={10}^{5}$.

Predefined mesh size	Domain elements	Boundary elements	$\overline{{\boldsymbol{Nu}}}$	|Ψ_*max*_|	CPU time (s)
Coarse	1102	122	0.1389	0.3066	10
Normal	1640	154	0.1383	0.3081	11
Fine	2418	192	0.1400	0.3086	12
Finer	7638	404	0.1396	0.3093	20
Extra fine	21332	788	0.1393	0.3096	47
Extremely fine	26830	788	0.1396	0.3098	57

## Results and Discussion

The reported results are set at $\varepsilon =0.7$, $Pr=4.623$ and $Le=3.5\times {10}^{5}$, ${N}_{BT}=1.1$, $Sc=3.55\times {10}^{4}$. These parameters represents the porosity, Prandtl number, Lewis number, normalized temperature, ratio of Brownian to thermophoretic diffusivity and Schmidt number. The important parameters in this study are: the portion of heated surface ($5 \% \le H\le 100 \%$), the nanoparticle volume fraction ($0.0\le \phi \le 0.04$), the Darcy number, ${10}^{-5}\le Da\le {10}^{-2}$ and the cylinder size, ($0.15\le R\le 0.25$).

**Figure 3:**
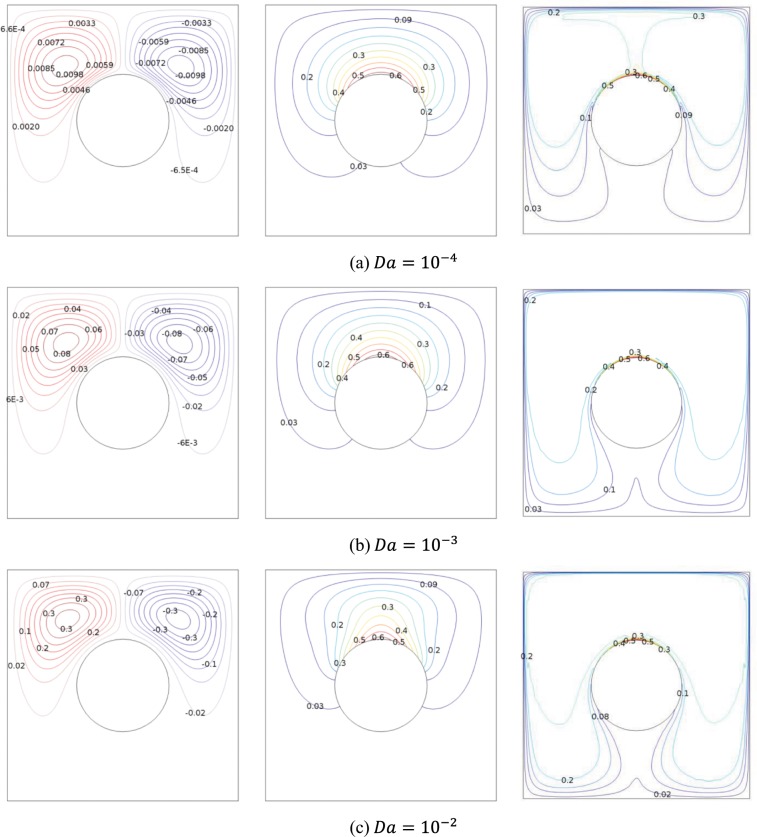
Effects of the heat source length on streamlines, isotherms and nanoparticles distribution at $R=0.2$, $\varphi =0.03$, $H=50 \%$ and $Da={10}^{-2}$.

Figure 3 illustrates the evolution of the streamlines, isotherms and isoconcentration by increasing the Darcy number at $R=0.2$, $\varphi =0.03$, $H=50 \%$ and $Da={10}^{-2}$. When the liquid temperature adjacent to localized heated surface rises where the hot liquid has lower density than the cold liquid. This creates a rotational flow, an anti-clockwise circulation cell in the left portion and clockwise circulation cell in the right portion. At small Darcy number, both of the flow circulation are slow and while at larger Darcy number, both of the flow circulation are moving quickly and the solid particle disperse wider with denser boundary layer at the bottom wall. It may be due to the fact that at high Darcy numbers where porosities enlarge, more particles are diffused within recirculating zones and accordingly, more deposition happen especially in the zone under the cylinder. Non-uniform shear rate leads to the movement of particles toward the central line. The reason is that thermophoretic force exerts on the particles in the contrary to the heat transport direction. The direction of heat transport is from the center of the active surface toward the wall. Thus, thermophoresis causes the particles migrate toward the center line.

**Figure 4:**
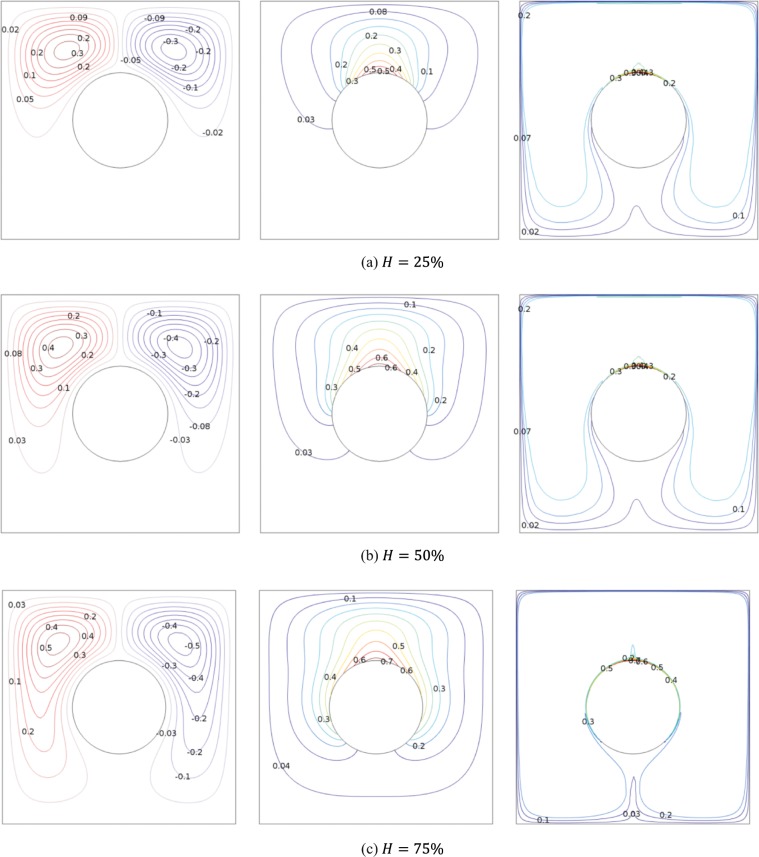
Effects of the heating size on streamlines, isotherms and nanoparticles distribution at $R=0.2$, $\varphi =0.03$ and $Da={10}^{-2}$.

Figure 4 illustrates the evolution of the streamlines, isotherms and isoconcentrations by increasing the heater size at $R=0.2$, $\varphi =0.03$ and $Da={10}^{-2}$. The streamlines show that as the *H* increases, the two overall rotating eddies are intensified. The cells are elongated vertically following increasing the heat source length. This occurs because of the larger density different are generated from the larger heated segment. Here the density variation intensify the flow circulation and circularize the isotherm. There is no isotherms below the cylinder for the case $H\le 50 \%$. The liquid temperature begins to distribute at the lower part for 75% heater flux size of the cylinder perimeter where denser nanoparticle distribution is also observed. Here, the nanoparticles movement happen in the central part below the cylinder. It is also noted that symmetrical circulating cells, isotherms and nanoparticle distribution were found for the considered heated segment size. The distribution of nanoparticles concentration is high at the upper cylinder surface, top wall and the upper half of the left and right wall. The thermophoresis acts against the gradient of temperature and moves the nanoparticles from upper cylinder surface to top wall and the upper half of the left and right wall. At the same times, the Brownian force discharges on the particles against the concentration gradient direction, where the thermophoresis and Brownian forces are directly facing each other. The Brownian force decreases while the shear rate effect increases. This brings a higher nanoparticles distribution at the active surface for a given mean concentration.

**Figure 5:**
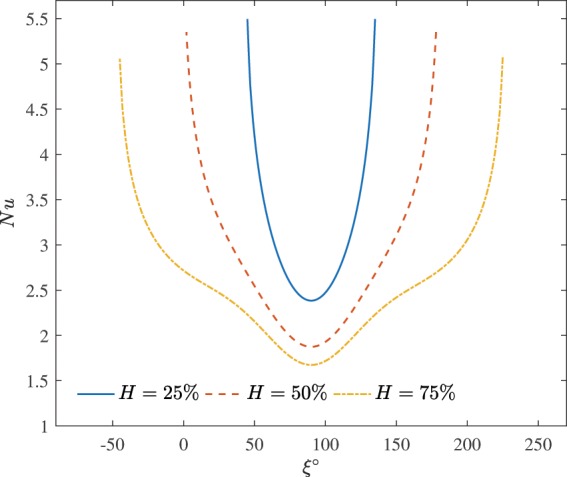
Effects of the heater size on the local Nusselt number at $R=0.2$, $\varphi =0.03$ and $Da={10}^{-2}$.

Figure 5 illustrates the influence of the heater size on the local Nusselt number at $R=0.2$, $\varphi =0.03$ and $Da={10}^{-2}$. This figure represent the heat transfer rate along the constant heated with heat flux and the insulated segment. The local heat transfer performance is minimum at $\xi =90^\circ$. The local heat transfer rate decreases while the portion of the heater increases. Reducing the length of the heater has locally elevated the thermal energy transport, namely, for $H=75 \%$ one can find non parabolic curve of the local Nusselt number profile, while further decreasing of *H* leads to close form of the parabolic curve.

**Figure 6:**
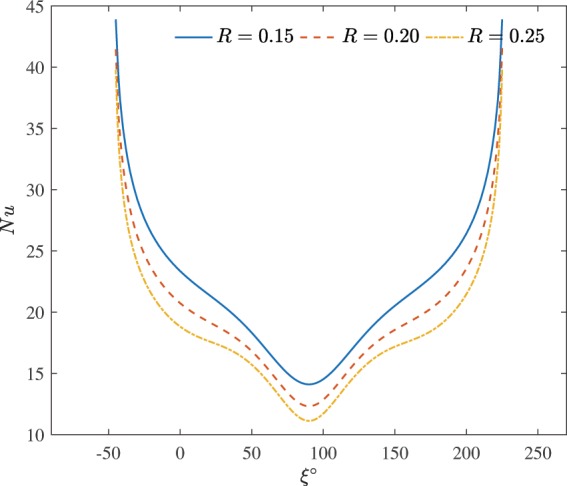
Effects of the cylinder size on the local Nusselt number at $H=75 \%$, $\varphi =0.03$ and $Da={10}^{-2}$.

Figure 6 illustrates the effect of the cylinder size on the local Nusselt number at $H=75 \%$, $\varphi =0.03$ and $Da={10}^{-2}$. The minimum heat transfer rate of the active surface were obtained at location $\xi =90^\circ$. The $R=0.25$ generate the lowest value of the local minimum. This minimum initiate from the symmetrical cold surfaces applied at left and right walls. The contrary cell motion prohibit direct heat transfer between the left and right cells. Each cell block the liquid movement from the opposite side. When the cylinder size is made smaller, the most importance response in temperature and nanoparticle concentration and then the local Nusselt number profiles is witnessed at the active surface.

**Figure 7:**
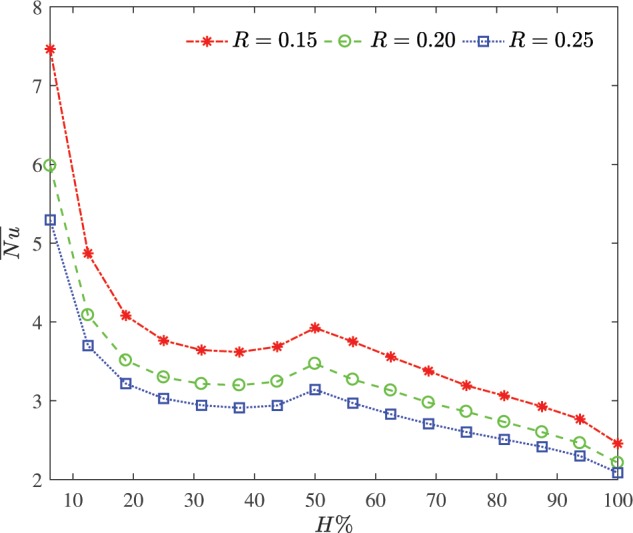
Effects of the cylinder size on the average Nusselt number versus the heater size at $\varphi =0.03$ and $Da={10}^{-2}$.

Figure 7 illustrates the effect of the cylinder size on the average Nusselt number versus the heater size at $\varphi =0.03$ and $Da={10}^{-2}$. Obviously, the smaller cylinder radius the higher average Nusselt number at the considered heater size. The the ratio of the heat transfer per unit area tends to decrease by increasing length portion of the heat source for the considered cylinder size. A rise of constant heat flux segment at $H=50 \%$ brings an extension of the thermal plume and fairly large heating of the cylinder surface. Simultaneously, the measurable amount of the bottom convective cells rises and magnitudes of region with high solution reduce. The cumulative influence is to increase the heat transfer rate significantly.

**Figure 8:**
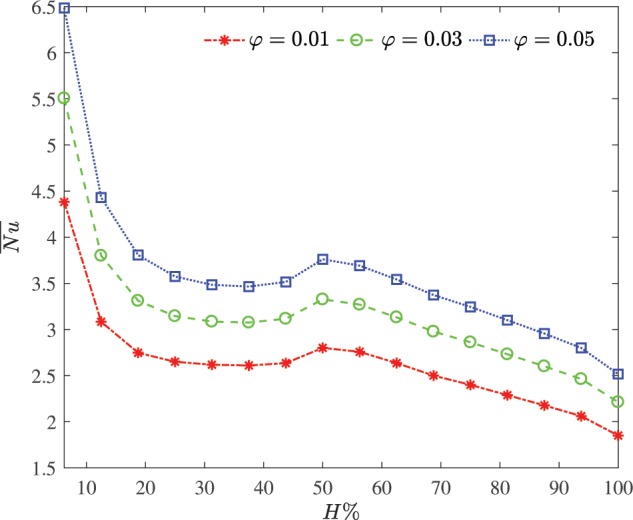
Influence of the average nanoparticles concentration on the average Nusselt number versus the heater size at $R=0.2$ and $Da={10}^{-2}$.

Figure 8 illustrates the influence of the average nanoparticles concentration on the average Nusselt number versus the heater size at $R=0.2$ and $Da={10}^{-2}$. The higher average nanoparticles concentration, the higher average Nusselt number at the considered heater size. When the thermally active surface increases, more heat is transferred into the system and thus the temperature in the annulus increase. In general, the ratio of the heat transfer per unit area of the heat source decreases as the length of the constant heat flux surface increases. The heat transfer rate is intensified for the half thermally active surface. This due to faster spreading of the thermal plume occurs at half thermally active surface. At the 50% heating portion, there is a local maximum in the Nusselt number values. The maximum Nusselt number accompany the stronger conductive nanoliquid at the sheet which rises the heat transfer performance at the sheet. This allows the heat enters deeper into the quiescent water. A growth of the heat transfer indicate linear variations of the average Nusselt number by varying the heater size with constant heat flux.

**Figure 9:**
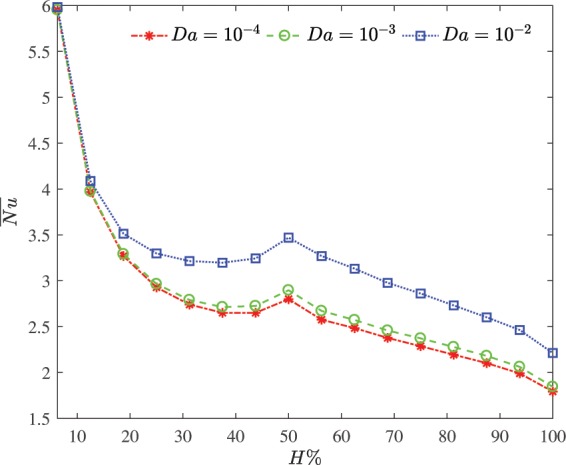
Influence of the Darcy number on the average Nusselt number versus the heater size at $\varphi =0.03$ and $R=0.2$.

Figure 9 illustrates the influence of the Darcy number on the average Nusselt number versus the heater size at $\varphi =0.03$ and $R=0.2$. Darcy number is beyond 10^−4^, the flow equations reduce to Darcy model. The nanoliquid particle moves with greater velocity for the high value of Darcy number and the velocity is modified at relative short of the heated surface. So that the enhancement in heat transfer by adjusting the porous medium permeability is more pronounced at $H > 20 \%$. At low values of the Darcy number, the nanoparticle movement is slow. This due to a retardation in the existence of solid fibers and decreasing in thermal conduction heat transfer within the porous medium. Deviation from the Darcy model increases the effectiveness of the nanoparticles in terms of heat transfer enhancement. At the current situation, it is important to use the heterogeneous nanoliquid model due to considerably effect of thermophoretic.

## Conclusions

The two-phase Buongiorno nanoliquid model was applied to formulate the continuity, momentum, energy and nanoparticles equations in dimensionless form, set of partial differential equation. Plotting of the local and average Nusselt numbers is given and the contour results of isotherms, streamlines and isoconcentrations within the enclosure are exhibited. Some important finding from this simulation are given below:

1. The streamlines, isotherms and nanoparticles isoconcentrations are symmetrical about the length of the enclosure. A higher nanoparticles distribution were obtained at the active surface for a given mean concentration.
2. A non parabolic curve of the local Nusselt number profile were found when the active surface portion greater than 50%. The minimum heat transfer rate of the active surface were obtained at location $\xi =90^\circ$.
3. In general, the ratio of the heat transfer per unit area of the heat source decreases as the length of the heated surface increases. The heat transfer rate is intensified for the half thermally active surface and high value of Darcy number. The higher average nanoparticles concentration, the higher heat transfer rate.
